# G‐CSF as a potential early biomarker for diagnosis of bloodstream infection

**DOI:** 10.1002/jcla.23592

**Published:** 2021-11-01

**Authors:** Huimin Li, Zhen Wang, Xuehua Li

**Affiliations:** ^1^ Department of Infectious Disease Jiaozhou People's Hospital Jiaozhou China; ^2^ Department of Infectious Disease Jiaozhou Renmin Hospital Jiaozhou China

**Keywords:** biomarker, bloodstream infection, G‐CSF

## Abstract

**Background:**

Cytokines play an important role in bacterial infection, and thus, we aim to find out cytokines that may be diagnostically significant in early stage of bacterial bloodstream infection.

**Methods:**

Mice models infected with *Staphylococcus aureus* and *Klebsiella pneumoniae* were established. Then dynamic changes of nine serum cytokines were monitored within 48 hours after the infection. Cytokines with significant differences between the infected groups and control group were further analyzed. Clinical samples of patients who were suspected of bloodstream infection were collected. Then the diagnostic efficiency of screened cytokines was determined with receiver operating characteristic curve analysis.

**Results:**

As for mice models infected by *Staphylococcus aureus* and *Klebsiella pneumoniae*, six cytokines including IL‐1β, IL‐6, IL‐12p70, G‐CSF, IFN‐γ, and TNF‐α were significantly different (*P* < .05) between two bacterial infected groups. As for clinical samples, three cytokines including IL‐6, IL‐12p70, and G‐CSF showed significant differences between infection group (*Staphylococcus aureus* and *Klebsiella pneumonia* group) and negative control group. With the area under curve of 0.7350 and 0.6431 for G‐CSF and IL‐6, respectively, these two cytokines were significantly different between *Staphylococcus aureus* and *Klebsiella pneumoniae* infection groups. Combination of G‐CSF and IL‐6 could improve the AUC to 0.8136.

**Conclusions:**

G‐CSF cannot only identify bacterial bloodstream infection, but can also distinguish the infection of *Staphylococcus aureus* from *Klebsiella pneumoniae*. Further investigation should be performed concerning the diagnostic efficiency of G‐CSF in diagnosing different types of bacterial bloodstream infection.

## Graphical abstract

Clinical samples of patients who were suspected of bloodstream infection were collected. Then, the diagnostic efficiency of screened cytokines was determined with receiver operating characteristic curve analysis. IL‐1β, IL‐6, IL‐12p70, G‐CSF, IFN‐γ, and TNF‐α were significantly different between two bacterial infected groups. G‐CSF cannot only identify bacterial bloodstream infection, but can also distinguish the infection of *Staphylococcus aureus* from *Klebsiella pneumoniae*.

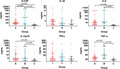

## 1 INTRODUCTION

Bacterial bloodstream infection (BSI) is an important cause of serious morbidity and mortality. Blood cultures are regarded as the “gold standard” for detection of BSI, but the turnaround time takes 3‐5 days. Furthermore, the sensitivity of blood culture decreases significantly when antibiotic therapy is initiated before blood samples are taken,^1^, ^2^ or when pathogens are fastidious or slow growing.^3^ Although Matrix Assisted Laser Desorption Ionization Time‐of‐Flight Mass Spectrometry (MALDI‐TOF MS) has been used in clinical laboratories to shorten the time required for microbial identification, it still requires the formation of bacterial colony and would also take more than 2 days to generate positive results. BSI progresses rapidly, which leads to the fact that clinicians prefer to apply empiric broad‐spectrum antimicrobial agents. Unfortunately, this practice contributes to the occurrence of multidrug‐resistant pathogens and increases the economic burden of patients simultaneously.^1^, ^2^ Therefore, we are in urgent need of fast and reliable diagnostic tools to provide precise rapid treatment and to enable de‐escalation antimicrobial treatment.^4^ Thus, we have to explore efficient biomarkers at the early stage of BSI.

When pathogen enters the bloodstream, the innate immune response is activated by recognition of pathogen‐associated molecule pattern (PAMP) such as peptides, lipopolysaccharide, and teichoic acid on the surface of bacteria.^5^ The pattern recognition receptor (PRR) of innate immune cells subsequently transmits the signal to the nuclear, promoting or inhibiting the release of inflammatory cytokines. Therefore, inflammatory cytokines are potential candidates as early diagnostic markers of bacterial infection. Up until now, several bacterial infection markers including white blood cell (WBC), C‐reactive protein (CRP), interleukin‐6 (IL‐6), vascular endothelial growth factor (VEGF), macrophage inflammatory protein‐1β (MIP‐1β), procalcitonin (PCT), tumor necrosis factor‐α (TNF‐α), IL‐2, IL‐8, and IL‐10 have been considered as the potential biomarkers for BSI diagnosis.^6^, ^7^

In order to screen for diagnostic markers of bloodstream infection for clinical application, 32 cytokines and chemokines related to the bacterial infection were chosen as potential parameters in clinical samples from patients. In addition, we aimed to further investigate the diagnostic biomarkers, which could distinguish gram‐negative bacteria from gram‐positive bacteria using mouse BSI model. Thus, mice infected with *Staphylococcus aureus* or *Klebsiella pneumonia* was used as the animal model to monitor the dynamic variation pattern of several infection‐related cytokines.

## 2 METHODS AND MATERIALS

### 2.1 Establishment of mice model of blood stream infection

#### 2.1.1 Preparation of bacteria strains

The standard strains *Staphylococcus aureus* ATCC25923 and *Klebsiella pneumoniae* ATCC 700603 were donated by the Department of Clinical Laboratory Medicine from our hospital. Two bacterial strains were incubated in LB medium (Oxide Microbiology Products, England) for 16‐18 hours at 37°C. Then the resuspended bacterial solution was formulated according to McFarley turbidity and diluted to different concentration gradients.

#### 2.1.2 Determination of half of the lethal dose 50 (LD50)

In order to investigate the dynamic changes of cytokines, mice model were established to simulate the process of bloodstream infection in vivo. Two strains of bacteria (*Staphylococcus aureus* and *Klebsiella pneumoniae*) were cultured at 37°C in 3 mL LB liquid medium, respectively. Then bacterial suspension was prepared and quantified by Maxwell turbidity method. The resuspended bacteria were first diluted to 3.9 Maxwell's turbidity. Then we performed a 10‐fold dilution and diluted six concentrations sequentially. The diluted bacterial suspension was injected into the mice through tail vein, 5 mice in each group, with 6 groups in total. The injection volume was 0.1 mL/10 g. Mice behaviors, weight changes, and death rate were observed and recorded daily for consecutive 7 days. The LD50 was calculated by Karber method.

#### 2.1.3 Establishment of mice model with bloodstream infection

To ensure that mice were alive under the infection condition during observation and blood collection, we chose 1/2 LD50 as the dose of bloodstream infection. Then we used clinical observation and molecular biological detection to determine the state of infection. A total of 105 specific‐pathogen‐free (SPF) male ICR mice, 6‐8 weeks old, were purchased from the Weitonglihua Experimental Animal Science and Technology Co., Ltd. [SCXK (Jing) 2018‐0011], weighing between 27 g and 33 g (the weight among different groups were not significantly different). They were fed in a SPF facility with the temperature of 18‐25°C and humidity of 50%‐70%. The experimental protocol was approved by the Animal Care and Use Committee of our hospital. All experiments were performed in accordance with relevant institutional and national guidelines and regulations.

In brief, the mice were inoculated with the 1/2 LD50 diluent of standard strain of *S aureus* or *K pneumoniae* in a final volume of 0.1 mL/10 g via tail veins. The time of challenge was designated as time 0 of the experiment. Blood from mice eye was collected 1, 3, 6, 12, 24, and 48 hours after inoculation and placed at 4°C for 8 hours to isolate the bacteria. Five mice were set for each time point. The establishment of bloodstream infection mice model was performed twice to validate the results.

### 2.2 Collection of clinical samples

In this study, blood samples were collected from the Department of Clinical Laboratory Medicine of Jiaozhou People's Hospital from January 2018 to October 2019. Blood samples of patients were collected before antibiotics treatment and also for blood culture. Serum samples were separated immediately with the centrifuge (Thermo‐Electron Corporation) at 1500 *g* for 5 minutes. Then the supernatant was transferred to polypropylene tubes (Solarbio, Beijing, China) and stored at −80° refrigerator before analysis.

Inclusion criteria: Patients with positive blood culture results, with PCT > 0.5 mg/mL or IL‐6 > 5.9 mg/mL or CRP > 0.8 mg/mL were included as BSI group. While patients with negative blood culture, with PCT < 0.5 mg/mL, IL‐6 < 5.9 mg/mL, and CRP < 0.8 mg/mL were recruited as negative control. Exclusion criteria: patients infected with multiple types of bacteria were removed.

### 2.3 Measurement of the serum cytokine levels, C‐reactive protein (CRP), and procalcitonin (PCT)

Serum levels of cytokines including IL‐1β, IL‐5, IL‐6, IL‐7, IL‐12p40, IL‐12p70, G‐CSF, IFN‐γ, and TNF‐α were detected using the Luminex^®^ xMAP™ System (Millipore Corporation, Germany). The assays were conducted in strict accordance with the operating instructions of the instrument. CRP and PCT were measured during the first 48 hours after onset of fever. Serum CRP was measured through nephelometric method (Siemens BNII, Cardiophase, Germany) and PCT was measured by Cobas800 (Roche, Switzerland) based on electrochemical luminescence technology.

### 2.4 Identification of bacteria from blood of mice after model establishment

Blood samples were collected for culture at the time point of 1, 3, 6, 12, 24, and 48 hours after injection. After inoculation on blood plates for 24 hours, single colony was picked for extracting DNA of bacteria. one colony of each type strain and clinical isolate was suspended in 100 μL DNA extraction solution (M&D, Wonju, Republic of Korea). The suspended bacterial solution was boiled for 10 minutes. After centrifugation at 13 000 *g* for 10 minutes, the supernatant was used as the DNA template. The real‐time PCR amplification was performed in a total volume of 20 μL containing 10 μL of 2 × Thunderbird probe quantitative PCR mixture, 5.0 μL of primer and TaqMan probe mixture, and 5 μL of template DNA; distilled water (DW) was added for a final volume of 20 μL. The primers used for *S aureus* were: Coa F: cct caa gca act the gaa aca aca, Coa R: tga atc ttg gtc tcg ctt cat and for *K pneumoniae* were fimH2 F: acg tgg tgg tcc cca cc, fimH2 R: tgc cga tga tcg act gca. Each run was under the following conditions: 95°C for 3 minutes and 35 cycles of 95°C for 20 seconds and 60°C for 40 seconds in a single real‐time PCR assay.

### 2.5 Statistical analysis

The Kolmogorov‐Smirnov or Shapiro‐Wilk tests were used to assess the normality of distribution of parameters. Continuous variables were described using the median (inter‐quartile range [IQR]) for non‐normally distributed data or the mean (standard error [SE]) for normally distributed data. Comparisons of group differences for continuous variables were made by the Mann‐Whitney *U* test or the Student *t* test as appropriate. Receiver operating characteristic (ROC) curves and areas under the curves (AUCs) were calculated to evaluate the performance of each biomarker for infection. The optimal cutoff values were set for each ROC curve through the Youden Index. Logistic regression was then performed to further investigate the diagnostic efficiency of combination of cytokines together. The values *P* < .05 were considered statistically significant. Statistical analysis was done using SPSS v. 22.0 (software SPSS Inc). All graphs were made by GraphPad Prism 7.0 software.

## 3 RESULTS

### 3.1 LD50 of *Staphylococcus aureus* and *Klebsiella pneumonia*

The number of dead mice and the survival rates for each concentration were recorded and calculated (Table S1). Then Karber method^8^ was used to determine the LD50 of the *S aureus* and *K pneumoniae*: 8.1 × 10^8^ CFU/mL and 1.11 × 10^8^ CFU/mL, respectively.

### 3.2 Clinical symptoms and changes of weight after infection in mice

**Figure 1:**
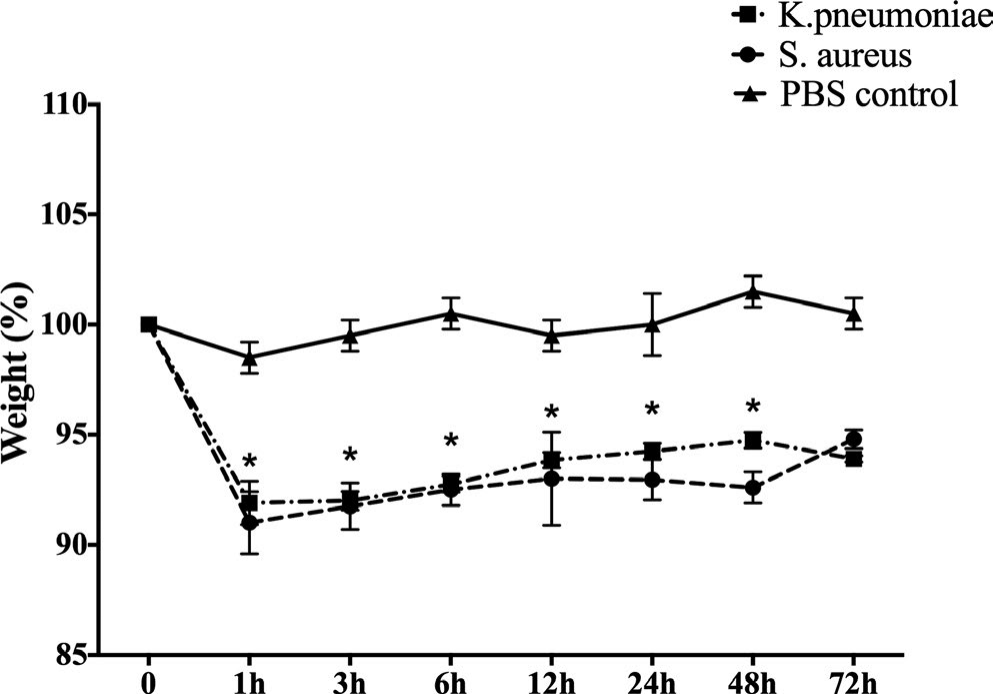
Changes of the body weight of mice models infected with *Staphylococcus aureus* and *Klebsiella pneumonia* or control group. The values were presented as percentage (n = 10 for each group). *P < .05, there were significant differences between infected group and control group

About 1 hour after the injection of bacteria, the mice in the experimental group showed symptoms of piloerection, closed eyes, and decreased activity. Three hours after injection, these behaviors were more obvious, with their bodies curling up and defecated loose stool. Twenty‐four hours after injection, mice had significantly decreased diet and activities. Forty‐eight hours after injection, death of mice occurred while the remaining alive ones returned to normal condition. In addition, weight of mice in *S aureus* group and *K pneumoniae* group decreased by an average of 3.9 g and 4.7 g, respectively 24 hours after infection when compared with the original weight. The weight of mice in both infected groups maintained at low levels during the first 48 hours and then gradually recovered 72 hours after infection (Figure 1).

### 3.3 Confirmation of bacteria from bloodstream infection mice models

**Figure 2:**
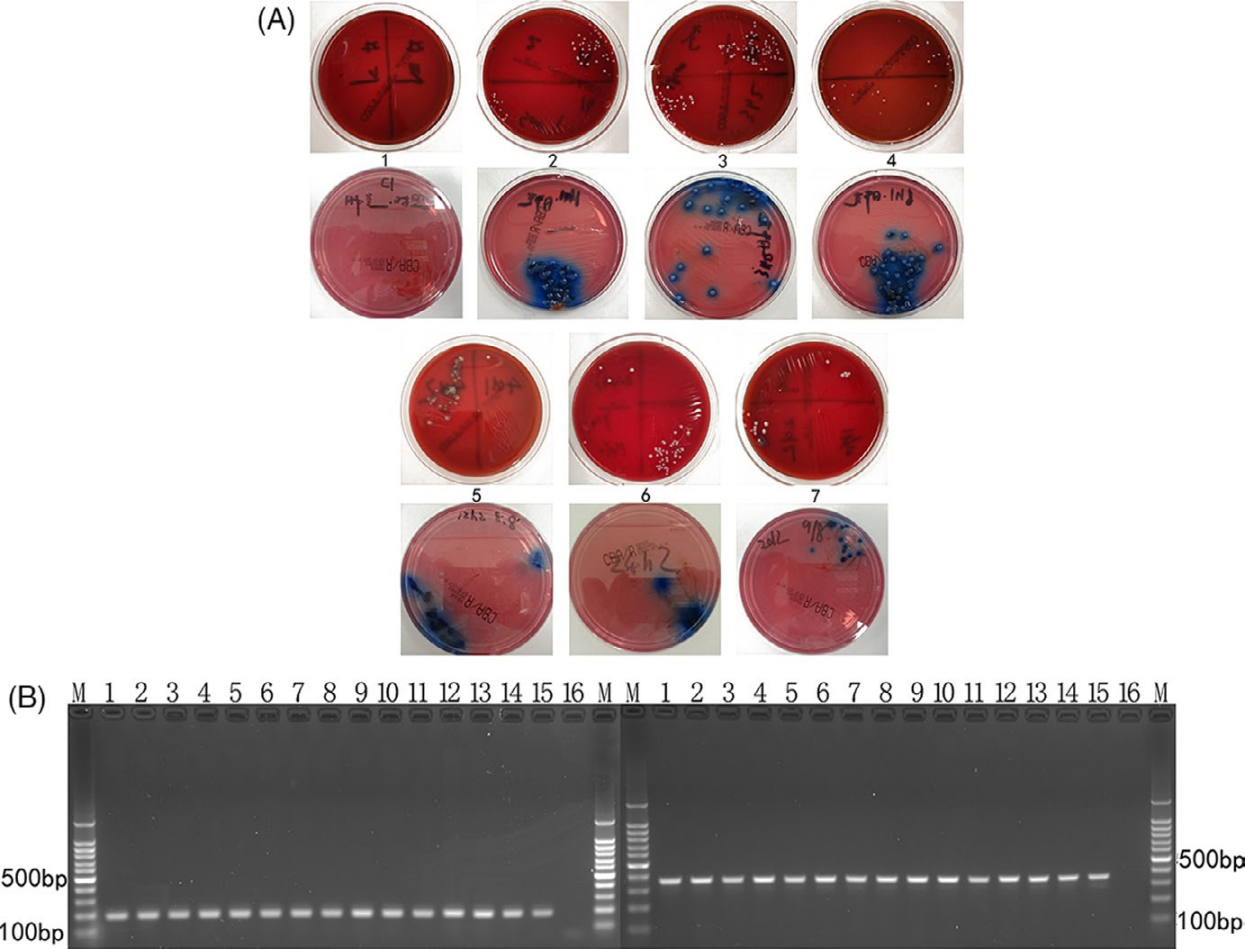
Confirmation of *Staphylococcus aureus* and *Klebsiella pneumonia* in infected groups. A, The results of blood culture at different time points after injection of bacteria in mice models. A1. The plate represented the blood culture result of control group. A2‐7. The plates represented the blood culture results of mice infected with *Staphylococcus aureus* or *Klebsiella pneumonia* at different time points (0.5 h, 3 h, 6 h, 12 h, 24 h, and 48 h after injection respectively). B, Electrophoretic results after PCR using extractions from blood after infection at different time points. M represented the ladder marker. Lane 1‐14 represented the results of 0.5 h, 1 h, 3 h, 6 h, 12 h, 24 h, and 48 h after infection of *Staphylococcus aureus* and *Klebsiella pneumonia*, respectively. Lane 15 and 16 represented the positive control and negative control, respectively

Figure 2A indicated the bacterial colony of *S aureus* and *K pneumoniae,* all of which were isolated from blood of mice models. After amplification, electrophoresis results were shown in Figure 2B.

### 3.4 Kinetic changes of nine cytokines in *S aureus* and *K pneumoniae* mouse infection models

**Figure 3:**
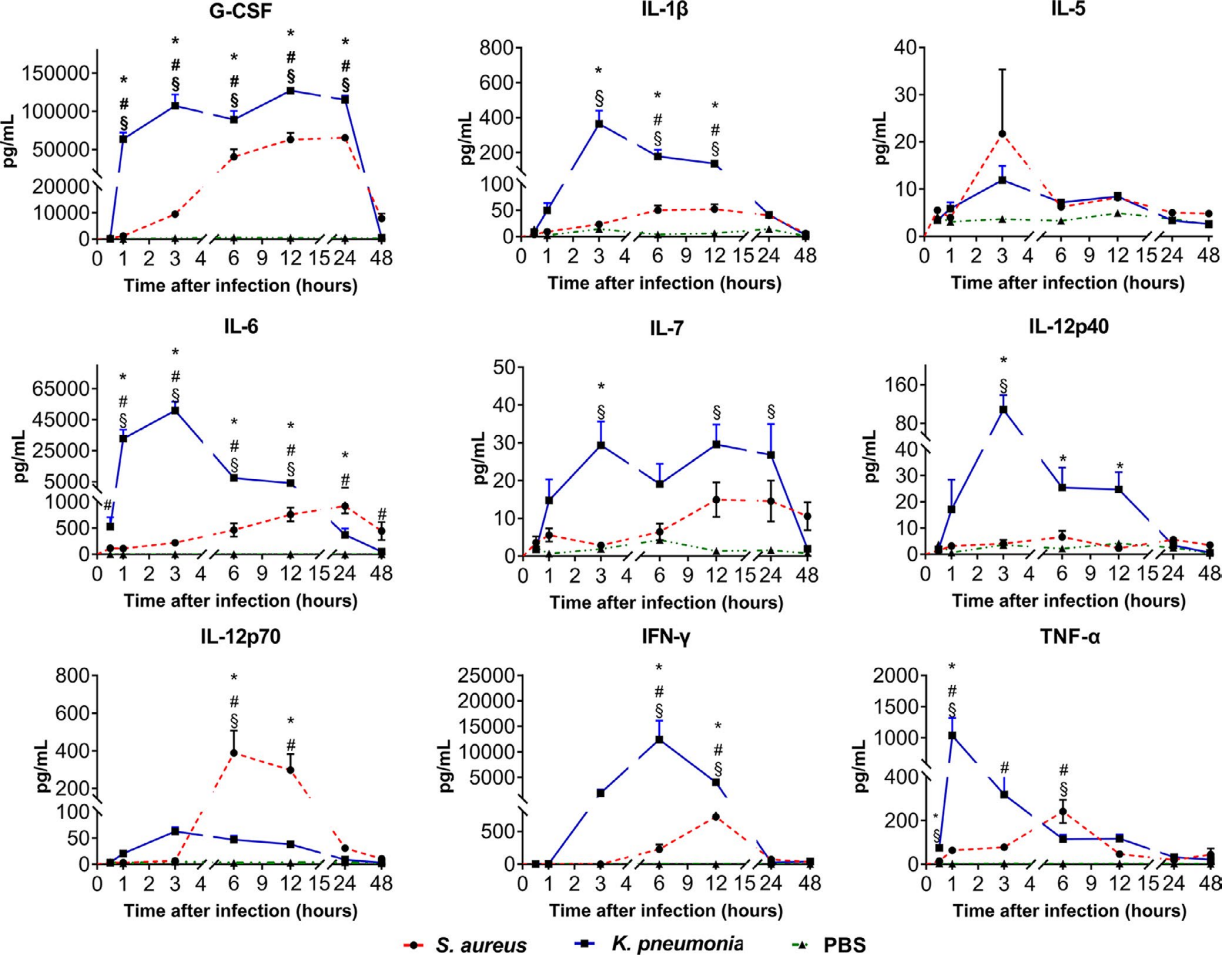
The kinetic changes of nine cytokines in the serum of infected mice models among *Staphylococcus aureus*, *Klebsiella pneumoniae* and negative control groups. *P < .05 comparing *S aureus* group with negative control group. **#** *P* < .05 comparing *K pneumoniae* group with negative control group. **§** *P* < .05 comparing *S aureus* group with *K pneumoniae* group

Kinetic changes of nine cytokines in serum collected from the three groups mice between 0 to 48 hours after injection are shown in Figure 3. All cytokines started to increase 0.5 hour after injection and decreased back to their initial level at 48 hours after reaching the highest peaks. The rate and quantity of the cytokines increased in *K pneumonia* group were higher than that of *S aureus* group after infection except for IL‐12p70. Among the 9 cytokines, the substantial change of G‐CSF is the same as IL‐6 between 0.5 and 6 hours. After 6 hours, IL‐6 dropped rapidly whereas G‐CSF still remains high. There were no significant changes for the levels of cytokines in control group during the experiment.

### 3.5 Comparison of six cytokines in serum samples between mice models infected *with S aureus* and *K pneumoniae*

We defined 0‐6 hours as the early stage of the infection after bacterial injection in mice models. During this stage, the statistical differences were found for G‐CSF between two infected groups at 1, 3, and 6 hours. For IL‐6, the difference occurred at 0.5, 1, 3, and 6 hours. For TNF‐α, the difference was at 1 hours, while for IL‐12p70, IFN‐γ, and IL‐1β, it was at 6 hours (*P* < .05). However, for IL‐5, IL‐7 and IL‐12p40, there was no statistical difference at any time point (*P* > .05). 48 hours after mice were infected with bacteria, the dynamic changes of inflammatory cytokines (such as G‐CSF) were remarkably different between infectious groups and non‐infectious group. There were also differences between the group of gram‐positive bacteria and the group of gram‐negative bacteria. However, other cytokines did not show the same difference.

### 3.6 General characteristics of enrolled patients

We then used clinical serum samples to further validate the diagnostic efficiency of these selected six cytokines. Among the 110 included patients, 67 patients were infected by *S aureus*, 23 infected by *K pneumoniae* and the other 20 patients had negative blood culture results. The age, sex, and clinical diagnosis were summarized in Table 1. Commonly used diagnostic biomarkers in clinical laboratory including CRP and PCT were not statistically different between *S aureus* group and *K pneumoniae* group. As for *S aureus* group, mean level of CRP and PCT were 11.01 ± 4.86 mg/dL and 1.98 ± 0.56 ng/mL, respectively. As for *K pneumoniae* group, mean level of CRP and PCT were 10.36 ± 3.79 mg/dL and 2.34 ± 0.51 ng/mL, respectively. While CRP and PCT in both gram‐positive bacterial group and gram‐negative bacterial group were significantly higher than those in negative control group (CRP: 0.56 ± 0.21 mg/dL, PCT: 0.35 ± 0.11 ng/mL).

**Table 1:** Clinical characteristics of clinical patients infected with *Staphylococcus aureus* and *Klebsiella pneumoniae* and negative control group

	*S aureus* group (n = 67)	*K pneumoniae* group (n = 23)	Negative control (n = 20)	*P* value
Age	58.9 (36‐79)	56.4 (34‐80)	51.2 (38‐60)	NS
Male/Female	36/31	13/10	11/9	NS
Primary diagnosis [n (%)]				
Urinary system infections	2 (2.9%)	4 (17.3%)	–	<.05 *
Cardiovascular system disease	5 (7.5%)	1 (4.3%)	–	NS
Obstructive jaundice or Biliary tract infection	6 (9.0%)	2 (8.7%)	–	NS
Hepatic disease	1 (1.5%)	2 (8.7%)	–	<.05
Abdominal pain or related symptoms	4 (6.0%)	2 (8.7%)	–	NS
Cancer	5 (7.5%)	2 (8.7%)	–	NS
Nervous system disease	3 (4.5%)	0	–	<.05
Musculoskeletal system disease	7 (10.4%)	1 (4.3%)	–	<.05
Respiratory system disease	19 (28.4%)	5 (21.7%)	–	NS
Dermatitis	2 (2.9%)	0	–	<.05
Endocrine system disease	2 (2.9%)	1 (4.3%)	–	NS
Gynecological disease	5 (7.5%)	1 (4.3%)	–	NS
Heat shock	0	1 (4.3%)	–	NS
Trauma	6 (9.0%)	1 (4.3%)	–	<.05

Note

Indicate a significant statistical difference between *S aureus* group and *K pneumoniae* group.

### 3.7 The cutoff values and diagnostic efficiencies for the potential biomarkers for bacterial bloodstream infection

**Figure 4:**
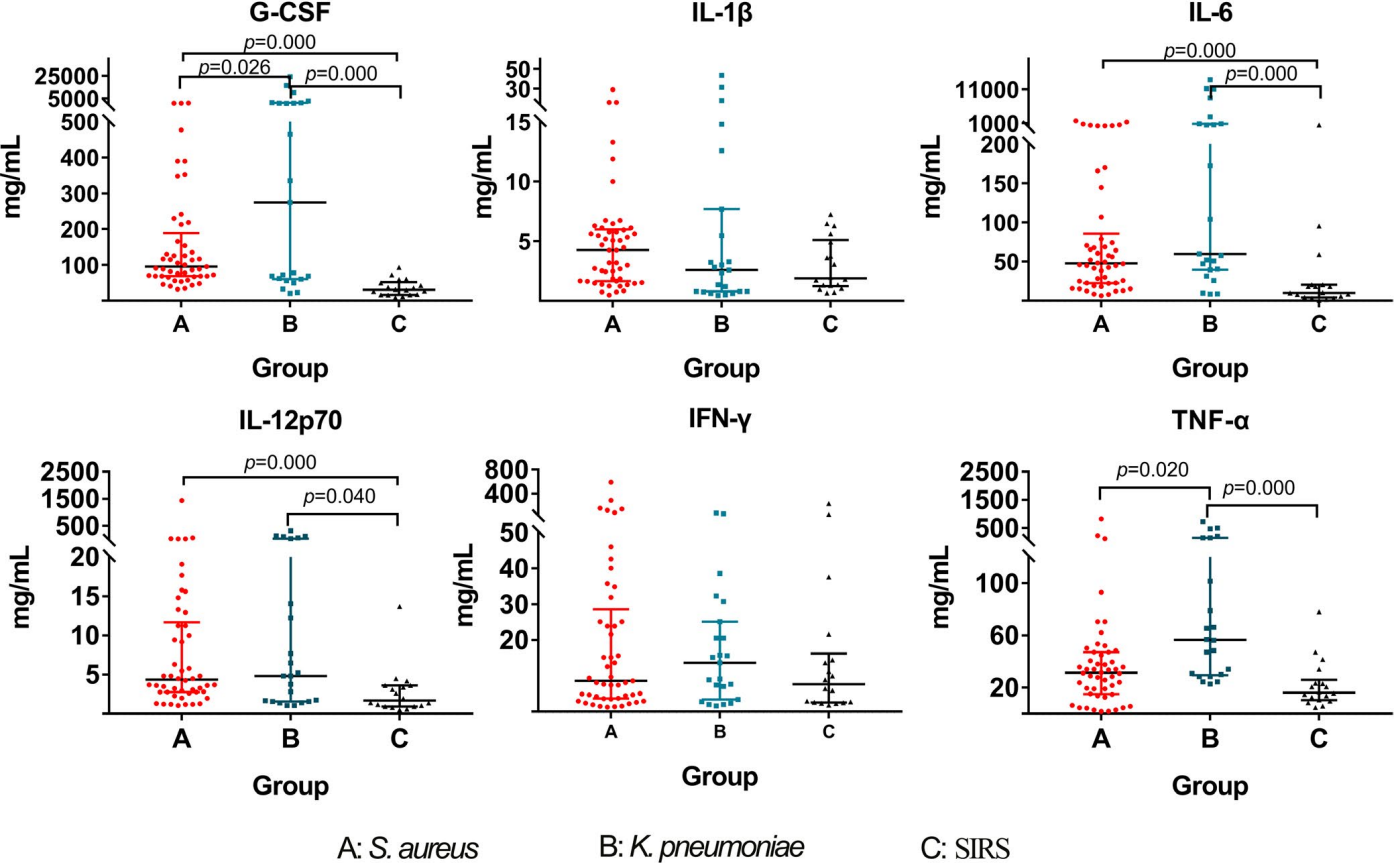
Changes of six cytokines in clinical serum samples among the infection of *Staphylococcus aureus, Klebsiella pneumoniae* and negative control groups. A, *S aureus* group. B *K. pneumonia* group. C. Negative control group

**Figure 5:**
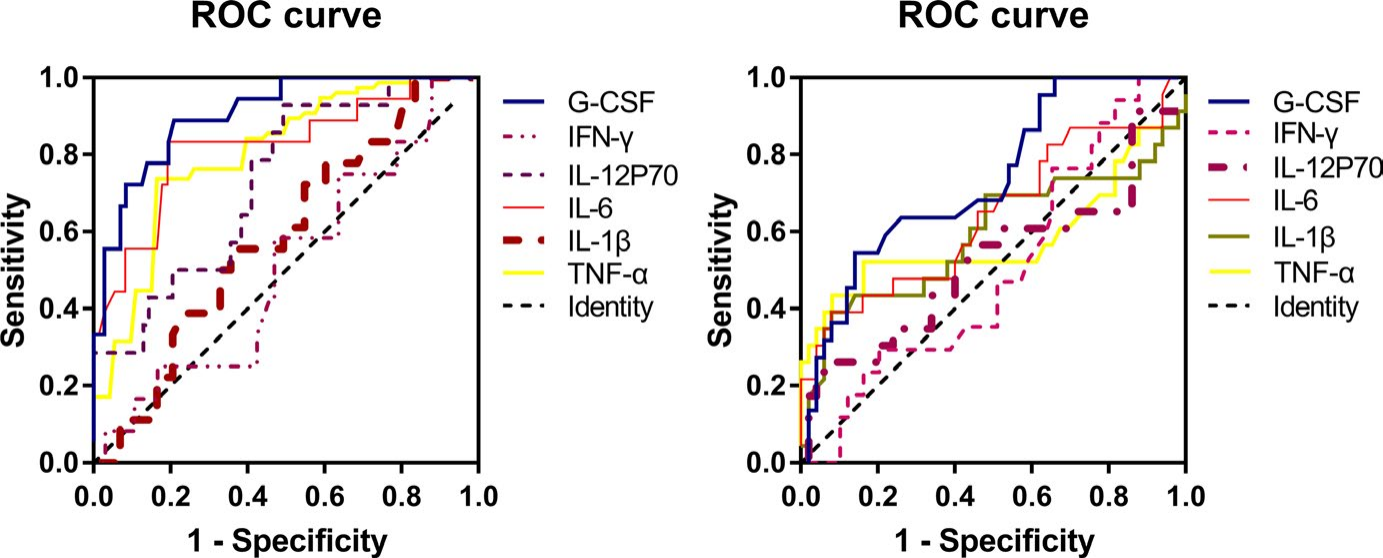
ROC curves for six cytokines in distinguishing between bloodstream bacterial infection and negative control (A) and between *Staphylococcus aureus* and *Klebsiella pneumoniae* infection (B)

The results of six cytokines (IL‐1β, IL‐6, IL‐12p70, G‐CSF, IFN‐γ, and TNF‐α) were shown as mean ± standard deviation and the differences among the *S aureus* group, *K pneumoniae* group and negative control group were shown in Figure 4. The ROC curves of IL‐1β, IL‐6, IL‐12p70, G‐CSF, IFN‐γ, and TNF‐α for differentiating between bacterial bloodstream infection and negative control were shown in Figure 5A. The AUCs calculated from the ROC curves were 0.9051 (95% Confidence interval (CI): 0.8375‐0.9767, *P* < .0001) for G‐CSF, 0.8227 (95% CI: 0.7026‐0.9427, *P* < .0001) for IL‐6, 0. 7309 (95% CI: 0.5959‐0.8659, *P* = .0064) for IL‐12p70, 0.6875 (95% CI: 0.5478‐0.8272, *P* < .0270) for TNF‐α, 0.5875 (95% CI: 0.4500‐0.7250, *P* = .2519) for IL‐1β and 0.5069 (95% C: 0.3339‐0.6800, *P* = .9393) for IFN‐γ. The cutoff values, sensitivities, and specificities for these six cytokines in bacterial bloodstream infection were shown in Table 2.

**Table 2:** Cutoff values and diagnostic efficiencies for potential biomarkers in bacterial bloodstream infection

	Cutoff (mg/mL)	Sensitivity (95% CI)	Specificity (95% CI)	Area under curve (95% CI)
G‐CSF	61.44	0.8889 (0.6529‐0.9862)	0.7917 (0.6798‐0.8784)	0.9051 (0.8375‐0.9767)
IL‐12p70	4.65	0.9286 (0.6613‐0.9982)	0.4932 (0.3740‐0.6128)	0. 7309 (0.5959‐0.8659)
IL‐6	21.38	0.8333 (0.5858‐0.9642)	0.8082 (0.6992‐0.8910)	0.8227 (0.7026‐0.9427)
IL‐1β	16.54	0.8762 (0.6782‐0.9345)	0.7892 (0.5983‐0.8987)	0.5875 (0.4500‐0.7250)
IFN‐γ	18.37	0.7758 (0.6294‐0.9345)	0.8023 (0.6934‐0.9237)	0.5069 (0.3339‐0.6800)
TNF‐α	23.63	0.7143 (0.4190‐0.9161)	0.7500 (0.6340‐0.8446)	0.6875 (0.5478‐0.8272)

The ROC curves of IL‐1β, IL‐6, IL‐12p70, G‐CSF, IFN‐γ, and TNF‐α for distinguishing *S aureus* or *K pneumoniae* infection were shown in Figure 5B. The AUCs calculated from the ROC curves were 0.7350 (95% CI: 0.6118‐0.8582) for G‐CSF and 0.6431 (95% CI: 0.4938‐0.7888) for IL‐6, whereas other four cytokines failed to differentiate the two types of bacterial infections. After logistic regression, the AUC of combining G‐CSF and IL‐6 was 0.8136 (95% CI: 0.6984‐0.8792) (Table 3).

**Table 3:** The Area under curve (AUC) for six cytokines in distinguishing between S. aureus and K.pneumoniae infection

	AUC	95% CI	*P* value
G‐CSF	0.7350	0.6118‐0.8582	.0259
IL‐6	0.6413	0.4938‐0.7888	.0536
IL‐12p70	0.5248	0.3673‐0.6823	.7350
TNF‐α	0.5816	0.4110‐0.7523	.2665
IL‐1β	0.5922	0.4308‐0.7535	.2081
IFN‐γ	0.5006	0.3466‐0.6546	.9942
G‐CSF + IL‐6	0.8136	0.6984‐0.8792	.0229

## 4 DISCUSSION

In recent years, the morbidity of sepsis caused by bacteria infection has increased.^9^ Lack of timely diagnosis and initial control, together with drug‐resistance due to overuse of antibiotics and persistent dysbacteriosis can lead to fatality.^1^, ^2^ Therefore, early diagnosis of bacterial infections in patients is of great significance. Specific and sensitive biomarker for early diagnosis of bacterial infection remains a challenge in current medicine, though many novel biomarkers were constantly being reported.

In the early stage of bacterial infection, the organism mainly produces innate immune response. The surface protein of bacteria can be combined with the pattern recognition receptors (PRRs) of immune cells, and the transcription and expression of inflammatory factors can be activated through cell signal transduction pathways such as MyD88.^10^ Due to the difference in the cell wall composition of gram‐negative and positive bacteria, the innate immune response caused by two types of bacteria is different, and thus the inflammatory factors vary. Therefore, we aimed to explore the changes of different cytokines in gram‐positive and gram‐negative bacterial bloodstream infection, to provide clinicians with more detailed diagnostic information. Zarkesh et al^11^ considered that IL‐6, CRP, WBC, and absolute neutrophil counts have diagnostic value to predict serious bacterial infection. Du et al^12^ explored interleukin 35 (IL‐35) as a novel candidate biomarker to diagnose early‐onset sepsis. Presepsin (sCD14‐ST)^13^, ^14^ has also been considered as a biomarker for infection. Therefore, it is essential to investigate a novel panel of biomarkers for bacterial infection.^15^, ^16^

Among all these biomarkers for infection, interleukin 6 (IL‐6) is thought to be more widely used in clinical laboratories. IL‐6 is an interleukin that acts as both a pro‐inflammatory cytokine and an anti‐inflammatory one,^17^ which is secreted by T cells and macrophages to stimulate immune response. Thus, it plays an important role in fighting infection and is often used clinically as an inflammatory biomarker. In our current study, the efficiency of IL‐6 is consistent with the previous literature,^11^ which could discriminate between infectious condition and non‐infectious condition.

Interestingly, our results also showed that the changing tendency of G‐CSF is not only similar to IL‐6, but also increased more significantly than IL‐6 in infected mice models.

G‐CSF reached its peak rapidly 1 hour after infection and this increase lasted for 24 hours, then it rapidly descended to the initial level at 48 hours. The changes of G‐CSF in *S aureus* infected group were more slightly when compared with *K pneumoniae* infected group (*P* < .01). This might be attributed to the more wild response of mice to *S aureus* infection than *K pneumonia*. G‐CSF is a glycoprotein that stimulates bone marrow to produce granulocytes and stem cells, which are then released into the bloodstream.^18^ G‐CSF can be produced by endothelium, macrophages, and immune cells from different tissues. It could not only act as a cytokine but also a hormone, and stimulates the survival, proliferation, differentiation, and function of neutrophil precursors and mature neutrophils. Based on its changes in our current study, we consider that G‐CSF could also be used as an infectious biomarker.

Changes of cytokines IL‐1β, IL‐5, IL‐7, and IL‐12p40 within 6 hours after infection in the animal model were not significantly different between the two types of bacteria infected groups. These results led us to suspect if the variance was caused by different concentration of bacteria injected between *S aureus* and *K pneumoniae*. However, the dynamics curve of IL‐12p70 dispelled the hypothesis. IL‐12p70 of mice infected with the same dose of *S aureus* was significantly higher than that of *K pneumonia*. Thus, we speculated that this might be related to the bacterial surface structure. IL‐12p40 and IL‐12p70 belong to different subunits of IL‐12. The performance of these two cytokines in this experiment shows the influence between them in the opposite manner.^19^, ^20^ The trend of IFN‐γ and TNF‐α in this animal model shows that they respond more slowly to bacterial infections than other cytokines.

In screening validated cytokines, we aimed to find biomarkers that could discriminate infected and non‐infected patients, as well as gram‐negative and positive bacteria, in the early stage of infection. We selected six cytokines that differed among the 3 groups of mice within 6 hours of post‐infection in the animal model. Then clinical validation results showed that IL‐12p70, G‐CSF, and IL‐6 had significant differences in the infection and non‐infection population. Although TNF‐α can distinguish between *S aureus* group and *K pneumoniae* group, it cannot differentiate between *S aureus* group and negative control group. However, G‐CSF was able to differentiate among negative control group, *S aureus* group and *K pneumonia* group. In addition, from the ROC curves that distinguish between infected and non‐infected groups, AUROC of G‐CSF was the highest among the six cytokines (AUC = 0.9051), which was superior to the IL‐6 (AUC = 0.8227). This also indicates that G‐CSF is significantly different among both infected groups.

There are still some limitations in our current study. Firstly, as for clinical samples, patients with CRP > 6.0 mg/mL or PCT > 0.8 mg/mL or IL‐6 > 5.9 mg/mL were excluded even when the culture results were negative. Since the positive rate of clinical blood culture was only 10%‐20%, although some blood cultures are negative, some may still be infected. Secondly, change of these nine cytokines in the animal model reflects the body's kinetic changes after bacterial infection. In the mouse model, with the progression of infection time, the level of cytokines is also undergoing major changes. However, the clinical samples from patients are taken at a single, specific time point, and we do not know exactly when the patient is infected. Therefore, it was not possible to accurately compare the results obtained from the mice model and the patients. Nevertheless, our statistical analysis can only reflect the changes of inflammatory factors in the patients after infection to a certain extent.

## 5 CONCLUSIONS

Based on the kinetic changes of these nine cytokines in animal models and the validation of six cytokines in clinical samples, G‐CSF is considered as a potential biomarker for early diagnosis of bloodstream infection and also as a possible index between *Staphylococcus aureus* and *Klebsiella pneumoniae* bloodstream bacterial infection. We will further detect the changes of inflammatory cytokines in other common clinical gram‐positive and gram‐negative bacteria bloodstream infections in the future study and also apply more clinical samples to further validate their diagnostic efficiencies.

## Supporting information

Table S1
