# Supplementary material for: G‐CSF as a potential early biomarker for diagnosis of bloodstream infection
Source: J Clin Lab Anal. 2021 Nov 1;35(12):e23592. doi: 10.1002/jcla.23592 (PMC8649329; doi:10.1002/jcla.23592)
Supplement: Supplementary file 1 — Table S1 [file JCLA-35-e23592-s001.docx]

**Supplementary Table 1. LD50 of *Staphylococcus*** [***aureus***](file:///C:\Program%20Files%20(x86)\Youdao\Dict\7.2.0.0511\resultui\dict\?keyword=aureus) **and *Klebsiella pneumonia***

| Group | Concentration (mg/kg) | No. of mice per group | No. of death per group | Death rate (%) |
| --- | --- | --- | --- | --- |
| 1 | 1×10^8^ | 5 | 5 | 100 |
| 2 | 1×10^7^ | 5 | 4 | 80 |
| 3 | 1×10^6^ | 5 | 3 | 60 |
| 4 | 1×10^5^ | 5 | 2 | 40 |
| 5 | 1×10^4^ | 5 | 2 | 40 |
| 6 | 1×10^3^ | 5 | 0 | 0 |
